# How Epistemic Beliefs about Climate Change Predict Climate Change Conspiracy Beliefs

**DOI:** 10.3389/fpsyg.2025.1523143

**Published:** 2025-04-14

**Authors:** Linnea Nöth, Lysann Zander

**Affiliations:** Division of Empirical Educational Research, Institute of Education, Leibniz Universität Hannover, Hannover, Germany

**Keywords:** conspiracy beliefs, epistemic beliefs, distrust in science, political ideology, mediation, climate conspiracy beliefs, conspiracy theory, climate change

## Abstract

Belief in climate change conspiracy theories (CCCT) can undermine support for measures against climate change. In two studies, we therefore aim to gain a clearer understanding of the factors that contribute to CCCT. A significant factor associated with CCCT is distrust in science, which is also correlated with epistemic beliefs (EBs) (e.g., beliefs are about the nature of knowledge and the process of knowing). EBs influence how individuals respond to knowledge claims, address contradictory evidence, and integrate new information. We hypothesize that EBs are linked to belief in CCCT via distrust in science. To test this hypothesis, we conducted one correlational study and one experimental study (*n* = 404 and *n* = 104, both pre-registered). Study 1 found that participants were more likely to endorse climate-related conspiracy beliefs if they viewed climate knowledge as tentative, relied on intuition to understand climate change, and had weaker beliefs in the interconnectedness of climate knowledge and its reliance on experts and scientific sources. As anticipated, distrust in climate science significantly mediated the relationships between the EBs subscales and belief in CCCT. Additionally, political ideology moderated the effect of believing knowledge originates from experts and science on distrust in climate science — this effect was pronounced among participants identifying with the political center while being weaker among left-wing participants. In Study 2, we were unable to establish a causal relationship between certainty beliefs and belief in CCCT. In conclusion, we suggest that educational initiatives aimed at fostering EBs may reduce science distrust and conspiracy beliefs.

## 1 Introduction

To achieve the goal of limiting global warming to 1.5°C, which aims to mitigate the adverse effects of climate change, it is essential to reduce CO_2_ emissions drastically and immediately (IPCC, 2023). Fortunately, governmental policies aimed at reducing carbon emissions are significantly correlated with decreased CO_2_ emissions (Le Quéré et al., 2019). However, the implementation of such policies depends on a variety of factors, including a country’s resources, economic conditions, available technologies, and voter preferences (Cherp et al., 2018). In a global context where one in five people subscribes to the belief that “climate change is a hoax” (YouGov, 2021), believing in climate change conspiracy theories (CCCT) could substantially influence people’s intentions to engage in necessary actions against the harmful effects of climate change. A recent meta-analysis by Biddlestone et al. (2022) supports this assertion, revealing that belief in CCCT leads to denial of climate science and science in general, diminished concern for environmental issues, reduced intentions to engage in ecologically responsible behaviors, and reduced support for environmental policies. In addition, conspiracy beliefs can directly influence voting behavior. On the one hand, people who believe conspiracy theories may perceive voting as futile (Freeman et al., 2022). On the other hand, conspiracy beliefs can predict people’s actual voting decisions, transcending their political views (Jolley et al., 2022). Thus, conspiracy theories about climate change may contribute to a delayed response to the climate crisis, exacerbating its impact on ecosystems, economies, and societies worldwide. Therefore, it is crucial to examine the underlying factors and circumstances that contribute to the acceptance of CCCT. This study aims to identify psychological processes that lead to the belief in CCCT and to determine the conditions under which these processes occur. Specifically, we will examine the influence of epistemic beliefs (EBs) [i.e., beliefs about the nature of knowledge and knowing (Hofer and Pintrich, 1997)] about climate change and distrust in climate science on the endorsement of CCCT.

### 1.1 Correlates of belief in climate change conspiracy theories

Correlates and antecedents of belief in CCCT^1^ have been identified by psychologists, sociologists, and political scientists. Factors related to belief in CCCT include aspects such as demographic characteristics, as well as psychological and ideological influences. In a large-scale study with participants from the Pacific Northwest, Sarathchandra and Haltinner (2021b) identified several factors that differentiated CCCT believers from those who did not believe in such reports. They found that, compared to non-believers, CCCT believers tend to be male, older, more conservative, more religious, and have a higher level of education. The study also found that participants who believed in CCCT experienced fewer negative emotions about climate change (e.g., worry, dread, and sadness) compared to non-believers. Moreover, believers had lower levels of trust in climate scientists and the mainstream media but greater trust in fossil fuel companies than participants who did not believe in CCCT. Believers and non-believers also differed in the sources from which they obtained information about climate change. While believers relied more on weather forecasts and the Fox News channel (a conservative news outlet), non-believers retrieved their information from (climate) scientists and news outlets such as MSNBC, CNN, or NPR, which are considered mainstream or liberal-leaning (Sarathchandra and Haltinner, 2021b). Moreover, belief in CCCT is linked to other types of conspiracy ideation. Those who believe in CCCT not only exhibit a *general tendency* to explain events through conspiracy theories (i.e., “conspiracy mentality”; Bruder et al., 2013; Bertin et al., 2021) but they are also inclined to endorse other *specific* conspiracy theories (e.g., those related to COVID-19; Freeman et al., 2022). Interestingly, belief in specific conspiracy theories that do not primarily focus on climate change is negatively related to empirically warranted, non-conspiratorial beliefs about the existence, origin, and impacts of climate change (Pan et al., 2022).

Similarly, rejecting climate science is predicted by both climate-specific conspiracy beliefs and conspiracy beliefs that are unrelated to science (e.g., about JFK) (Lewandowsky et al., 2013). Van der Linden (2015) explored the causal link between belief in CCCT and acceptance of climate science through an experiment. Participants were assigned to one of three groups: a ‘conspiracy’ group that watched a video about CCCT, a ‘pro-climate’ group that viewed a video about taking action against climate change, and a control group that did not watch a video. Participants in the conspiracy condition expressed significantly greater agreement with the statement that climate change would be a hoax compared to those in the control group. Additionally, participants in the conspiracy condition believed there was less consensus on human-made climate change compared to both the control group and the pro-climate group. These findings highlight that the acceptance or rejection of climate science plays a role in support for CCCT.

### 1.2 Trust in science and scientists

Another factor that has been meta-analytically linked to the belief in climate science is the extent to which people trust scientists (Hornsey et al., 2016). Distrusting scientists is not only related to skepticism about climate change but also associated with the endorsement of CCCT, as shown by Sarathchandra and Haltinner (2021b). However, the relationship between distrust in scientists and conspiracy beliefs is not limited to the topic of climate change. In several correlational studies, distrust in science or scientists was related to COVID-19 conspiracy beliefs (Tonković et al., 2021; Chayinska et al., 2022; Vranic et al., 2022), generic conspiracy beliefs about malevolent actions of governments, secret organizations, and extraterrestrial contact (Fasce and Picó, 2019), and conspiracy mentality in general (Nera et al., 2022). Recognizing the multiple links between conspiracy beliefs and distrust in science and scientists, we aim to examine the factors and conditions affecting these relationships. More specifically, we focus on the relationship between distrust in climate science and scientists and belief in CCCT. Because educational research has established a link between EBs and trust in science within different domains (e.g., Strømsø et al., 2011; Schoor, 2023), we investigate whether EBs are also related to belief in CCCT and whether this relationship is mediated by trust in climate science.

### 1.3 EBs as a source of science distrust and climate conspiracy beliefs

EBs are beliefs about the nature of knowledge and knowing (Hofer and Pintrich, 1997). According to Hofer and Pintrich’s (1997) framework, the term “nature of *knowledge*” refers to the question “*What* is knowledge?” while “nature of *knowing*” is reflected in the question “How does one *acquire* knowledge?” and is, therefore, more procedural. Beliefs about the nature of knowledge consist of beliefs about the *certainty* of knowledge and beliefs about the *simplicity* of knowledge. Beliefs about the “certainty of knowledge” (Hofer and Pintrich, 1997, p. 119) span from the belief that knowledge is absolute and does not change to the belief that knowledge is provisional and may change over time. Beliefs about the “simplicity of knowledge” (Hofer and Pintrich, 1997, p. 120) range from the view that knowledge consists of a collection of more or less isolated facts to understanding it as strongly interrelated concepts that may depend on their context. Beliefs about the nature of knowing comprise beliefs about the *source* of knowledge and the *justification* for knowing. Beliefs about the acquisition of knowledge (“source of knowledge,” Hofer and Pintrich, 1997, p. 120) range from the belief that knowledge is received from external authorities such as experts or scientists to the belief that knowledge is actively constructed through interaction with others. Beliefs about the justification basis of knowledge (“justification for knowing,” Hofer and Pintrich, 1997, p. 120) range from justifying claims relying on personal observation, authority, or intuition to justifying claims employing inquiry rules, evaluating different sources, and integrating them. EBs can change over time and get more sophisticated as a person’s level of education increases (King and Kitchener, 2001). Kuhn et al. (2000) propose a developmental perspective that describes the progression from rather low-level EBs to sophisticated ones. They argue that at the lowest levels, the realist and absolutist level, knowledge is seen as objective, certain, either right or wrong, and located in the external world. At the multiplist level, however, knowledge is seen as a product of human cognition and as provisional. At this stage, assertions are seen as subjective opinions, and all opinions hold equal value. In the last stage, the evaluativist level, knowledge is also seen as uncertain and created by humans. However, claims are viewed as “judgments” (Kuhn et al., 2000, p. 311), which can be verified and substantiated by evidence and arguments. Nevertheless, the degree of sophistication of beliefs about the nature of knowledge and knowing depends on the context, that is specific scientific topics and domains (Elby and Hammer, 2001; Sinatra et al., 2014). Although the notion of “tentative knowledge,” for example, is considered sophisticated (Kuhn et al., 2000), Sinatra et al. (2014) argue that it would not “be beneficial to an understanding of science to think that there is an ever-present, similar degree of uncertainty on every topic” (p. 127). Using climate change as an example, they explain that it is reasonable to recognize the tentativeness of scientific knowledge in general while *also* acknowledging the scientific consensus on human-induced climate change^2^. This context-specificity of EBs also has implications for the measurement of EBs (Bråten et al., 2009). A measure that takes context-specificity into account was introduced by Peter et al. (2016), who argued that sophisticated EBs are manifested in low endorsement of both generalized (i.e., not context-specific) absolutist and generalized multiplist statements.

### 1.4 EBs and conspiracy beliefs

EBs can influence how people deal with (every day) scientific information. Sinatra et al. (2014) suggest that EBs play a crucial role in assessing scientific claims, particularly when individuals encounter contradictory information and must evaluate it carefully. Moreover, EBs come into play when people have to incorporate new knowledge into existing knowledge or need to integrate knowledge from different sources. We propose that EBs might play a role when people are confronted with conspiratorial claims about scientific issues like COVID-19, genetically modified food, or climate change. This is because EBs have been related to common correlates of conspiracy beliefs, such as the need for closure (DeBacker and Crowson, 2006; Peter et al., 2016), the need for cognition (Nussbaum and Bendixen, 2003), and critical thinking (Muis et al., 2021). Also, EBs predict conspiracy beliefs. In an early study, Garrett and Weeks (2017) used another conceptualization of EBs with the dimensions “faith in intuition for facts,” “need for evidence,” and “truth is political.”^3^ They found that relying on one’s gut feeling when evaluating facts predicts stronger conspiracy beliefs (e.g., about the Moon landing or the assassination of JFK), as does the belief that facts and truth are determined by those in power. In contrast, valuing evidence predicts lower conspiracy beliefs. Moreover, participants with a higher need for evidence were more likely to believe that climate change is human-made, while participants who thought that the truth would be political were more likely to disbelieve this. The relationships between faith in intuition, the need for evidence, and the idea that truth would be political were replicated with respect to COVID-19-related conspiracy beliefs (Rudloff et al., 2022). Serrano et al. (2023) also explored the relationship between EBs and COVID-19-related conspiracy beliefs, following Peter et al.’s (2016) conceptualization of EBs. They showed that college students’ beliefs that knowledge is subjective, uncertain, and constructed by their own reasoning (i.e., multiplist beliefs) were positively linked to conspiracy beliefs about COVID-19. This relationship was mediated by trust in science. Multiplist beliefs were negatively related to trust in science, which, again, was negatively related to COVID-19 conspiracy beliefs. Consistent with Serrano et al. (2023), we expect that EBs about climate change will predict believing CCCT.

### 1.5 EBs and trust in science and scientists

Similar to the work by Serrano et al. (2023), other studies have also observed a connection between EBs and trust in science and scientists. Participants who believed knowledge was uncertain and preliminary and who relied on their intuition to justify knowledge claims were less likely to trust scientists and science in general (Schoor, 2023). In the same study, Schoor (2023) found that participants who justified knowledge claims based on authorities such as experts were more likely to trust in science and scientists. These findings are complemented by a study by Strømsø et al. (2011), which found that participants who believed that knowledge about climate change primarily originates from their own thinking and interpretations – rather than relying on expert statements – were less likely to trust a popular science text about climate change. In comparison, participants who believed knowledge about climate change should be justified by thinking critically and comparing multiple sources were more likely to trust this text. Moreover, faith in intuition when judging claims is negatively related to believing information from scientific and liberal sources compared to conservative ones (Butterfuss et al., 2020). Although their study did not find a direct link between EBs and trust in these sources, it suggested that EBs might be connected to political ideology. Building on these insights, we anticipate that EBs about climate change will be linked to distrust in climate science and will seek to examine whether political ideology also plays a role in the relationship between EBs and distrust in science.

#### 1.5.1 Influence of political ideology

A recent large-scale study with a sample of 67 countries on all inhabited continents revealed that conservatives tend to trust scientists less (Cologna et al., 2024; preprint). Yet this relationship varies greatly from country to country. While this relationship is negative for European countries, North America, Brazil, and Israel, it is non-existent in the majority of the countries – or even positive, for example, for countries in Eastern Europe, Southeast Asia, and Africa. In a sample of US students, Serrano et al. (2023) also found that distrust in science is linked to conservatism, whereas EBs showed no association with conservatism. Given the association between conservatism and distrust in science and the potential link between political ideology and EBs (Butterfuss et al., 2020), we examine whether political ideology moderates the relationship between EBs and distrust in science.

### 1.6 Hypotheses

We test the following hypotheses in one correlational study and one experimental study:

EBs about climate change will correlate with and predict belief in CCCT (H1). More specifically, we expect that certainty beliefs and acquisition beliefs positively correlate with and predict belief in CCCT. We expect that justification-based beliefs negatively correlate with and predict belief in CCCT. We also expect that simplicity beliefs correlate with and predict belief in CCCT.

Trust in climate science will mediate the relationship between EBs about climate change and belief in CCCT (H2).

Political ideology will moderate the relationship between EBs about climate change and trust in climate science (H3).

## 2 Study 1

### 2.1 Materials and methods

#### 2.1.1 Sample and design

We collected the data from 404 German-speaking participants (211 women, 184 men, and 4 others; *M*_age_ = 27.43 years, *SD* = 7.55 years). Thirty percent had a high school degree, and 59% had some kind of university degree (bachelor’s, master’s, PhD). Participants were recruited via flyers, an online bulletin board of a German university, and recruitment platforms like www.surveycircle.com or www.survey-swap.io. Data were collected using a German questionnaire on the LimeSurvey platform. The participants did not receive payment. We determined our sample size prior to sampling. According to Fritz and MacKinnon (2007), the required sample size for a mediation analysis using percentile bootstrapping for a small effect size in the alpha path (*f* = 0.14), a large effect size in the beta path (*f* = 0.59), and a power of 80% would be *N* = 398. The study was pre-registered: https://osf.io/mzrfh. Following the procedure suggested by Willroth and Atherton (2024), a table containing the deviations and extensions of the preregistration can be found at: https://osf.io/h9aby. The study was approved by the Central Ethics Committee of Leibniz University Hannover and the Hanover University of Music, Drama, and Media.

#### 2.1.2 Measures

##### 2.1.2.1 EBs about climate change and climate science

Participants were asked to rate 24 statements (12 reverse coded) about their EBs about climate change and climate science. The items share similarities with those of Bråten et al. (2009). However, we chose to create new items in order to align the item texts more strongly to the four dimensions introduced by Hofer and Pintrich (1997). Six items were designed for each sub-dimension (e.g., beliefs about certainty, simplicity, acquisition, and justification basis of knowledge). Participants rated the items using a 7-point Likert scale (1 = *strongly disagree*, 7 = *strongly agree*). An example of the measurement of beliefs about the certainty of knowledge would be the following item: “Knowledge about the climate is constantly changing.” The items were presented randomly. Reliability indices for each measurement can be found in Table 1.

**Table 1:** Correlations among measures (Study 1).

Variable	*α*	*M*	*SD*	1	2	3	4	5	6	7
1. Certainty Beliefs	0.64	4.97	1.22							
2. Structure Beliefs	0.64	5.85	0.95	0.04						
3. SASOKB	0.58	5.35	1.00	0.20***	0.08					
4. EASOKB	0.75	6.10	0.88	−0.05	0.36***	0.05				
5. Subjective Justification Beliefs	0.68	2.79	1.33	0.07	−0.32***	0.15**	−0.28***			
6. Political Ideology	/	2.96	1.18	0.10	−0.37***	0.06	−0.23***	0.15**		
7. Climate Science Distrust	0.91	2.36	1.01	0.15**	−0.49***	0.05	−0.46***	0.25***	0.51***	
8. Climate Conspiracy Beliefs	0.90	2.18	1.24	0.19***	−0.35***	0.10	−0.37***	0.15**	0.41***	0.74***

M and SD are used to represent mean and standard deviation, respectively. The symbol α represents the reliability coefficient of Cronbach’s alpha. *p < 0.05, **p < 0.01, ***p < 0.001. All correlations were examined using two-tailed significance tests. SASOKB refers to Self as a Source of Knowledge Beliefs; EASOKB refers to Experts as a Source of Knowledge Beliefs.

We tested the factorial structure of the scale using a Confirmatory Factor Analysis (CFA) and an Exploratory Factor Analysis (EFA). The results of the CFA and EFA can be found in the Supplementary material. The fit indices of the original four-factor structure of the scale were not acceptable. We, therefore, adjusted the model and yielded acceptable to good fit indices. The adjusted measure consisted of the following subscales: certainty of knowledge (three items), structure of knowledge (three items), self as a source of knowledge (three items), experts as a source of knowledge (three items), and subjective justification (three items).

##### 2.1.2.2 Distrust in climate science

Distrust in climate science was measured using the 14-item (Dis)Trust in Climate Science Scale by Sarathchandra and Haltinner (2021a); e.g., “Climate scientists ignore those who disagree with them”. Responses were given on a 7-point scale (1 = *strongly disagree*, 7 = *strongly agree*).

##### 2.1.2.3 Climate change conspiracy beliefs

We measured participants’ climate change-related conspiracy beliefs using six items by Bertin et al. (2021; e.g., “Some scientists falsify their results, concluding that climate change is due to humans, in order to gain power and influence.”). A 7-point scale (1 = *strongly disagree*, 7 = *strongly agree*) was used.

##### 2.1.2.4 Political ideology

To measure the participants’ political ideology, we used the Left–Right Self-Placement (ALLBUS) (Breyer, 2015). After reading the following instruction: “Many people use the terms ‘left’ and ‘right’ when they want to describe different political views. Here, we have a scale that runs from left to right. Thinking of your own political views, where would you place these on this scale?” Participants were asked to indicate their political ideology on a 7-point scale ranging from 1 “left” to 7 “right.”

#### 2.1.3 Procedure

After giving informed consent, participants filled out the scales measuring their EBs, distrust in climate science, and belief in CCCT and indicated their political affiliation. Before being thanked and debriefed, participants were asked to give their demographic information (age, gender, education) and were asked whether they had participated in the study seriously. The scales measuring EBs and distrust in climate science each included one attention check (“Please indicate ‘strongly disagree’” and “Please indicate ‘strongly agree’”).

#### 2.1.4 Statistical analyses

Mediation analysis was used to test hypotheses 1 and 2. To test hypothesis 3, a moderated mediation model was estimated. Before running the mediation analyses, we examined the regression assumptions (e.g., linearity, normality, homoskedasticity, no multicollinearity, no autocorrelation of errors, and absence of outliers).

We used the PROCESS model 4 in R to conduct the mediation analysis (Hayes, 2022). Due to non-normal data and heteroscedasticity, we used percentile bootstrapping for all model parameters (*n* = 5,000 bootstrap samples, seed = 654321) and heteroskedasticity-consistent standard errors (HC4). Besides reporting unstandardized effect sizes with percentile bootstrapped confidence intervals for all paths and the direct effect (*bs*), we also report completely standardized effect sizes (*Bs*).

Mediation and moderated mediation analyses were calculated for each sub-dimension of EBs (e.g., beliefs about the certainty of knowledge, the structure of knowledge, the self as a source of knowledge, experts as a source of knowledge, and the subjective justification of knowledge). In both models, the epistemic belief sub-dimension was the predictor variable, distrust in climate science was the mediator variable, and belief in CCCT was the criterion variable. In the moderated mediation, political ideology was added as a moderator variable for the a-path from EBs to distrust in climate science. The moderated mediation was conducted using the PROCESS model 7 with percentile bootstrapping for all model parameters (*n* = 10,000 bootstrap samples, seed = 654321). The variables were mean-centered, and conditional effects were estimated for the mean value of political ideology and values corresponding to 1 *SD* plus and 1 *SD* minus the value for the mean political ideology.

### 2.2 Results

The correlations between all variables can be found in Table 1.

#### 2.2.1 Beliefs about the certainty of knowledge (short: certainty beliefs)

A positive effect of certainty beliefs on belief in CCCT was observed, *B* = 0.189, *p* < 0.001 (*total effect*). Certainty beliefs predicted distrust in climate science, *b* = 0.133, 95% CI [0.064, 0.204] (*B* = 0.160, *p* < 0.001), which, in turn, predicted belief in CCCT, *b* = 0.921, 95% CI [0.834, 1.002], (*B* = 0.747, *p* < 0.001). Participants who perceived knowledge about climate change as uncertain were more likely to distrust climate science, which, in turn, increased their endorsement of CCCT. The relationship between certainty beliefs and belief in CCCT is partially mediated by distrust in climate science, *b* = 0.070, 95% CI [0.134, 0.127] (*B* = 0.069, *p* = 0.018) (*direct effect*). The indirect effect was also significant, *B* = 0.120, 95% CI [0.057, 0.183], suggesting mediation (Zhao et al., 2010).

The index of moderated mediation was not significant, *b* = 0.047, 95% CI [−0.005, 0.101]. For the a-path from justification beliefs to distrust in climate science, there was no significant interaction between justification beliefs and political ideology, *b* = 0.052, *p* = 0.082. The b-path from distrust in climate science to belief in CCCT was significant, *b* = 0.889, *p* < 0.001. The direct effect from certainty beliefs to belief in CCCT was, too, *b* = 0.074, *p* = 0.027.

#### 2.2.2 Beliefs about the structure of knowledge (short: structure beliefs)

A negative effect of structure beliefs on belief in CCCT was observed, *B* = −0.374, *p* < 0.001 (*total effect*). Structure beliefs negatively predicted distrust in climate science, *b* = −0.549, 95% CI [−0.648, −0.444] (*B* = −0.527, *p* < 0.001), which, in turn, predicted belief in CCCT *b* = 0.921, 95% CI [0.834, 1.002] (*B* = 0.770, *p* < 0.001). Participants who perceived knowledge about climate change as complex and interconnected were more likely to trust climate science, which subsequently reduced their endorsement of CCCT. The relationship between structure beliefs and belief in CCCT is fully mediated by distrust in climate science, *b* = 0.032, 95% CI [−0.073, 0.139] (*B* = 0.024, *p* = 0.567) (*direct effect*). This is also reflected in the significant indirect effect, *B* = −0.398, 95% CI [−0.470, −0.320].

The index of moderated mediation was not significant, *b* = −0.072, 95% CI [−0.151, 0.002]. However, the conditional indirect effect for participants identifying with the political center (+ 1 *SD*) was the strongest, *b* = −0.418, 95% CI [−0.557, −0.281], it was weaker for center-left participants (*M*), *b* = −0.333, 95% CI [−0.430, −0.237], and smallest for participants identifying as left (− 1 *SD*), *b* = −0.248, 95% CI [−0.374, −0.124]. For the a-path from structure beliefs to distrust in climate science, there was a significant interaction between structure beliefs and political ideology, *b* = −0.078, *p* = 0.029, *ΔR^2^* = 0.008. The conditional effect from structure beliefs on distrust in climate science was the strongest for participants identifying with the political center (+ 1 *SD*), *b* = −0.452, *p* < 0.001, it was weaker for center-left participants (*M*), *b* = −0.361, *p* < 0.001, and smallest for participants identifying as left (− 1 *SD*), *b* = −0.269, *p* < 0.001. The b-path from distrust in climate science to belief in CCCT was significant, *b* = 0.924, *p* < 0.001. The direct effect of structure beliefs on belief in CCCT was not significant, *b* = 0.027, *p* = 0.593.

#### 2.2.3 Beliefs about the self as a source of knowledge (short: self as a source of knowledge beliefs)

Self as a source of knowledge beliefs did not predict belief in CCCT, *B* = 0.085, *p* = 0.118 (*total effect*) and also did not predict distrust in climate science, *b* = 0.042, 95% CI [−0.073, 0.154] (*B* = 0.042, *p* = 0.461). However, distrust in climate science predicted belief in CCCT, *b* = 0.932, 95% CI [0.885, 1.009] (*B* = 0.756, *p* < 0.001). The direct effect of self as a source of knowledge beliefs on belief in CCCT via distrust in climate science was not significant, *b* = 0.066, 95% CI [−0.008, 0.140] (*B* = 0.053, *p* = 0.087). The indirect effect was also not significant, *b* = 0.032, 95% CI [−0.054, 0.115].

The index of moderated mediation was not significant, *b* = 0.040, 95% CI [−0.034, 0.120]. For the a-path from self as a source of knowledge beliefs to distrust in climate science, there was no significant interaction between self as a source of knowledge beliefs and political ideology, *b* = 0.044, *p* = 0.180. The b-path from distrust in climate science to belief in CCCT was significant, *b* = 0.909, *p* < 0.001. The direct effect of self as a source of knowledge beliefs to belief in CCCT was not significant, *b* = 0.075, *p* = 0.068.

#### 2.2.4 Beliefs about experts as a source of knowledge (short: experts as a source of knowledge beliefs)

A negative effect of experts as a source of knowledge beliefs on belief in CCCT was observed, *B* = −0.404, *p* < 0.001 (*total effect*). Experts as a source of knowledge beliefs negatively predicted distrust in climate science, *b* = −0.563, 95% CI [−0.679, −0.448] (*B* = −0.491, *p* < 0.001), which, in turn, predicted belief in CCCT, *b* = 0.910, 95% CI [0.805, 1.003] (*B* = 0.737, *p* < 0.001). Participants who perceived knowledge about climate change as originating from experts and scientists were more likely to trust climate science, which, in turn, decreased their endorsement of CCCT. The relationship between experts as a source of knowledge beliefs and belief in CCCT is fully mediated by distrust in climate science, *b* = −0.060, 95% CI [−0.169, 0.051] (*B* = −0.042, *p* = 0.291) (*direct effect*). This is also reflected in the significant indirect effect, *B* = −0.362, 95% CI [−0.433, −0.285].

The index of moderated mediation was significant, *b* = −0.098, 95% CI [−0.185, −0.016]. The conditional indirect effect for participants identifying with the political center (+ 1 *SD*) was the strongest, *b* = −0.446, 95% CI [−0.622, −0.323], it was weaker for center-left participants (*M*), *b* = −0.350, 95% CI [−0.454, −0.256], and smallest for participants identifying as left (− 1 *SD*), *b* = −0.234, 95% CI [−0.374, −0.117]. For the a-path from experts as a source of knowledge beliefs to distrust in climate science, there was a significant interaction between experts as a source of knowledge beliefs and political ideology, *b* = −0.111, *p* = 0.006, *ΔR^2^* = 0.012. The conditional effect from experts as a source of knowledge beliefs on distrust in climate science was the strongest for participants identifying with the political center (+ 1 *SD*), *b* = −0.525, *p* < 0.001, it was weaker for center-left participants (*M*), *b* = −0.394, *p* < 0.001, and smallest for participants identifying as left (− 1 *SD*), *b* = −0.264, *p* < 0.001. Put simply, the negative relationship between believing that climate change knowledge originates from experts and scientists and distrust in climate science was stronger for participants identifying with the political center compared to those with left-leaning views. The b-path from distrust in climate science to belief in CCCT was significant, *b* = 0.888, *p* < 0.001. The direct effect from experts as a source of knowledge beliefs to belief in CCCT was not significant, *b* = 0.098, *p* = 0.136.

#### 2.2.5 Beliefs about the subjective justification of knowledge (short: subjective justification beliefs)

A positive effect of subjective justification beliefs on belief in CCCT was observed, *B* = 0.159, *p* = 0.002 (*total effect*). Subjective justification beliefs predicted distrust in climate science, *b* = 0.196, 95% CI [0.127, 0.270] (*B* = 0.260, *p* < 0.001), which, in turn, predicted belief in CCCT, *b* = 0.949, 95% CI [0.855, 1.033] (*B* = 0.770, *p* < 0.001). Participants who believed knowledge about climate change should be justified through intuition, and personal observation were more likely to distrust climate science, which, in turn, increased their endorsement of CCCT. The relationship between subjective justification beliefs and belief in CCCT is fully mediated by distrust in climate science, *b* = −0.038, 95% CI [−0.105, 0.029] (*B* = −0.041, *p* = 0.264) (*direct effect*). This is also reflected in the significant indirect effect, *B* = 0.199, 95% CI [0.128, 0.273].

The index of moderated mediation was not significant, *b* = 0.003, 95% CI [−0.052, 0.058]. For the a-path from subjective justification beliefs to distrust in climate science, there was no significant interaction between subjective justification beliefs and political ideology, *b* = 0.003, *p* = 0.908. The b-path from distrust in climate science to the belief in CCCT was significant, *b* = 0.924, *p* < 0.001. The direct effect of subjective justification beliefs to belief in CCCT was not significant, *b* = −0.031, *p* = 0.332.

### 2.3 Discussion

In Study 1, we examined the relationships between EBs about climate change, distrust in climate science, and belief in CCCT. To measure EBs about climate change, we developed an *ad hoc* scale to measure the four dimensions of EBs by Hofer and Pintrich (1997: certainty, simplicity, source, justification of knowledge). However, a confirmatory factor analysis indicated that the four-factor model did not adequately fit the data and that a five-factor structure provided a significantly better fit. This five-factor structure consisted of the following dimensions: beliefs about the certainty of knowledge, structure of knowledge, the self as a source of knowledge, experts as a source of knowledge, and the subjective justification of knowledge. While the dimensions of certainty and structure (“simplicity” in the Hofer and Pintrich (1997) framework) remained unchanged, the source dimension was split up into two dimensions (i.e., the self vs. experts as a source of knowledge), and the justification dimension only comprised subjective justification. By distinguishing between self and experts as sources of knowledge, we recognize that individuals can simultaneously engage in personal research and rely on scientific authorities, allowing for a nuanced assessment of EBs that can be used to reveal effects that were previously less apparent. Moreover, we think that beliefs about the justification of knowledge are strongly connected to beliefs about the source of knowledge (Greene et al., 2008). Therefore, we believe that beliefs about experts as a source of knowledge about climate change may as well overlap with the justification by authority (i.e., experts or scientists) dimension that Greene et al. (2008) differentiated from personal justification.

Study 1 suggests that different views about climate change knowledge predict greater or lesser belief in CCCT. More specifically, participants who believed knowledge about climate change is provisional or who believed climate change knowledge is best grounded in gut feeling and personal observation endorsed stronger belief in CCCT. Conversely, participants who believed knowledge about climate change is interrelated and complex, as well as those believing experts and science are the main sources of knowledge about climate change, endorsed lower belief in CCCT. Distrust in climate science mediated all the relationships between the different EBs’ subscales and belief in CCCT. This suggests that different views about climate change knowledge affect one’s (dis-)trust in climate science, which, again, affects whether someone believes conspiracy theories about climate change. Contrary to our expectations, political ideology only affected the strength of the relationship between believing that experts were the main source of knowledge about climate change and distrust in climate science. Strikingly, the positive relationships between the EBs’ subscales and distrust in climate science did not depend on political ideology. This indicates that for these individuals, skepticism toward climate science may arise from perceptions of uncertainty and reliance on personal observations rather than from their political beliefs. Another explanation may be the limited variability of the political ideology scores that lay between 1.78 and 4.14 on a 7-point scale (*M* = 2.96, *SD* = 1.18), which may have led to a reduced power for detecting moderation (Memon et al., 2019). Finally, the new dimension, “self as a source of knowledge,” was neither related to distrust in science nor to belief in CCCT, potentially due to the low reliability of the subscale. Taken together, our results corroborate prior research that linked different conceptualizations of EBs to belief in conspiracy theories (Garrett and Weeks, 2017; Rudloff et al., 2022; Serrano et al., 2023). Similar to Serrano et al. (2023), we found that distrust in science mediates the link between EBs and conspiracy beliefs. By examining a different conspiracy narrative and using an alternative conceptualization of EBs, we found initial evidence for the robustness of this relationship. Moreover, our findings contribute to the evidence documenting the links between EBs and trust in science (Strømsø et al., 2011; Schoor, 2023) as well as between trust in science and conspiracy beliefs (Fasce and Picó, 2019; Tonković et al., 2021).

One limitation of our study was the partly low reliability of our *ad hoc* EBs measure, potentially leading to an underestimation of the relationships between EBs and belief in CCCT or distrust in climate science (Schmitt, 1996). Yet, despite the low reliability of the measure, we were able to establish moderate relationships between the EBs’ subscales, distrust in climate science, and climate-related conspiracy beliefs. One exception to this is the scale that was intended to measure the dimension of “Self as a source of knowledge.” It had poor reliability coefficients and only correlated with subjective justification beliefs. Moreover, our study was correlational, which is why we cannot draw causal conclusions. Therefore, we conducted a second, experimental study.

## 3 Study 2

In the second study, we chose an experimental approach to investigate the causal relationship between EBs, distrust in climate science, and CCCT. Therefore, we allocated participants to two experimental groups and one control group and measured their agreement to distrust in climate science and CCCT statements. As in Study 1, we also assessed participants’ political ideologies. Study 2 was also pre-registered: https://osf.io/btkq7. A table containing the deviations and extensions of the preregistration can be found at: https://osf.io/62rmg. The study was approved by the Central Ethics Committee of Leibniz University Hannover and the Hanover University of Music, Drama, and Media.

### 3.1 Materials and methods

#### 3.1.1 Sample and design

We collected a sample of *n* = 132 German-speaking Prolific workers, of whom *n* = 104 passed the manipulation check described below (54 women, 50 men; *M*_age_ = 30.46 years, *SD* = 10.40 years). 26% had a high school degree, and 63% had some kind of university degree (bachelor’s, master’s, PhD). Data were collected via LimeSurvey, and participants received £0.80 for their participation. However, due to budgetary constraints, our pre-registered sample size target had to be adjusted. Using the effect sizes obtained in Study 1, we found that, according to Fritz and MacKinnon (2007), a mediation analysis using percentile bootstrapping for small-to-medium effect size in the alpha path (*f* = 0.26), a large effect size in the beta path (*f* = 0.59), and a power of 80% would require a sample size of *N* = 122.

We used a between-subjects design where we randomly allocated participants to one of three conditions. The first experimental group read a short text intended to promote naïve EBs (72 words). The second experimental group was presented with a short text intended to promote rather sophisticated EBs (69 words). The control group did not receive a text. Both experimental texts show moderate readability (text level: fiction or non-fiction), according to the readability index Lix by Björnsson (1971) and can be found in the Supplementary material. Participants were asked to read the texts carefully. However, due to the restrictions of the LimeSurvey license our university holds, it was not possible to hide the “next” button. Thus, participants were able to skip the experimental manipulation. This is also reflected in their rather short reading times: Participants in the naïve beliefs condition had an average reading time of *M* = 22.43 s (*SD* = 13.08, with a minimum reading time of 4.21 s and a max reading time of 66.23 s). Participants in the sophisticated beliefs condition had a mean reading time of *M* = 34.83 s (*SD* = 62.46; Min = 3.05; Max = 410.79). There was no significant difference in reading time between the experimental groups: *F*(1, 73) = 1.290, *p* = 0.260. After the experimental manipulation, the participants filled out exactly the same measures used in Study 1, were asked which text they read (if they had been asked to read a text at the beginning of the questionnaire) and whether they read it thoroughly, and were debriefed. Participants in the experimental groups passed the manipulation check if they selected the correct text, whereas participants in the control group passed if they indicated that they were not asked to read a text.

#### 3.1.2 Measures

The measures were identical to the measures of Study 1.

#### 3.1.3 Analytic strategy

We followed the procedure for dealing with multi-categorical independent variables in mediation suggested by Hayes and Preacher (2014). First, we checked whether our experimental condition had a significant effect on participants’ belief in CCCT. Due to heterogeneous variances in the groups, we conducted the Kruskal–Wallis test instead of an ANOVA. Moreover, we checked whether our experimental manipulation had a significant effect on participants’ EBs subscales. Finally, we also examined if the manipulation had an effect on distrust in climate science. Depending on whether the assumptions for conducting an ANOVA were met, we either performed an ANOVA or the Kruskal–Wallis test. As in Study 1, mediation analysis was conducted using the PROCESS model 4 by Hayes (2022), while moderated-mediation analysis was conducted using the PROCESS model 7, both in R. The experimental groups were dummy-coded and treated as multi-categorial independent variables in both models.

### 3.2 Results

Descriptive statistics can be found in Tables 2, 3, and the correlations between the variables can be found in Table 4.

**Table 2:** Descriptive statistics of the variables for Study 2.

	Distrust in climate science (*Mediator*)		Climate conspiracy beliefs (*DV*)		Certainty beliefs		Structure beliefs		SASOKB		EASOKB		Subjective justification beliefs	
	*M*	*SD*	*M*	*SD*	*M*	*SD*	*M*	*SD*	*M*	*SD*	*M*	*SD*	*M*	*SD*
Naïve (*n* = 34)	2.30	0.88	2.32	1.32	4.81	1.23	5.55	0.91	5.23	1.04	6.37	0.68	3.00	1.54
Sophisticated (*n* = 41)	2.75	1.14	2.85	1.45	5.54	1.13	5.25	1.15	5.21	0.87	6.21	0.72	2.77	1.54
Control (*n* = 29)	2.56	1.16	2.47	1.34	5.14	1.06	5.30	0.99	5.17	1.16	5.93	0.94	2.61	1.29
All groups combined (*n* = 104)	2.55	1.07	2.57	1.38	5.19	1.17	5.36	1.03	5.20	1.00	6.18	0.78	2.80	1.37

M and SD are used to represent mean and standard deviation, respectively. n is used to denote the sample size. DV = “dependent variable”; SASOKB = Self as a Source of Knowledge Beliefs; EASOKB = Experts as a Source of Knowledge Beliefs.

**Table 3:** Sociodemographic characteristics of participants per experimental group.

Demographic variable	Naïve beliefs (*n* = 34)		Sophisticated beliefs (*n* = 41)		Control (*n* = 29)		ANOVA *F*(2,101)
	*n*	*%*	*n*	*%*	*n*	*%*	
Gender							
Female	19	56	18	44	17	59	0.885 (*n.s.*)
Male	15	44	23	56	12	41	
	*M*	*SD*	*M*	*SD*	*M*	*SD*	
Age	30.62	11.78	31.29	10.27	29.10	8.97	0.377 (*n.s.*)

*N = 104. n.s. indicates no significant group difference.*

**Table 4:** Correlations among measures (Study 2).

Variable	*α*	1	2	3	4	5	6	7
1. Certainty Beliefs	0.72							
2. Structure Beliefs	0.58	−0.02						
3. SASOKB	0.53	0.16	0.06					
4. EASOKB	0.67	0.04	0.38***	0.10				
5. Subjective Justification Beliefs	0.76	0.01	−0.43***	0.12	−0.45***			
6. Political Ideology	/	0.17	−0.26**	0.01	−0.18	0.28**		
7. Climate Science Distrust	0.92	0.20*	−0.42***	0.03	−0.56***	0.49***	0.48***	
8. Climate Conspiracy Beliefs	0.92	0.32**	−0.42***	−0.05	−0.47***	0.51***	0.40***	0.76***

α is used to represent the reliability coefficient of Cronbach’s alpha. *p < 0.05; **p < 0.01; ***p < 0.001. All correlations were assessed using two-tailed significance tests. SASOKB refers to Self as a Source of Knowledge Beliefs, while EASOKB denotes Experts as a Source of Knowledge Beliefs.

#### 3.2.1 Effect of the experimental manipulation

According to the Kruskal–Wallis test, the experimental groups did not differ with respect to their belief in CCCT, χ^2^(2) = 3.042, *p* = 0.218. The effect of the experimental manipulation was only significant for certainty beliefs: *F*(2, 100) = 3.81, *p* = 0.026. A *post-hoc* comparison revealed that participants in the naïve condition had significantly lower certainty beliefs than participants in the sophisticated condition (*p* = 0.021). However, participants in the control group differed neither from the naïve condition nor from the sophisticated condition. Simplicity beliefs were not affected by the experimental manipulation, χ^2^(2) = 0.891, *p* = 0.641. The same is true for self as a source of knowledge beliefs, χ^2^(2) = 0.460, *p* = 0.795, experts as a source of knowledge beliefs, χ^2^(2) = 3.894, *p* = 0.143, and subjective justification beliefs, χ^2^(2) = 1.421, *p* = 0.491. The experimental manipulation also did not influence participants’ distrust in climate science, χ^2^(2) = 3.304, *p* = 0.192.

#### 3.2.2 Mediation and moderated mediation

The omnibus test of the total effect of the three experimental conditions (naïve EBs, sophisticated EBs, and control group) on belief in CCCT was not significant, *F*(2,101) = 1.422, *p* = 0.246, indicating that the three groups did not differ in terms of their effect on climate conspiracy beliefs. Neither participants in the naïve condition (*B =* −0.249, *p* = 0.312) nor participants in the sophisticated condition (*B = 0*.169, *p* = 0.517) differed from the control group in terms of their distrust of climate science. These findings indicate that the naïve and sophisticated treatment conditions did not significantly influence participants’ levels of distrust in climate science compared to the control group. However, distrust in climate science predicted belief in CCCT, *B = 0*.765, *p* < 0.001. Moreover, the direct effect of the experimental conditions on belief in CCCT was not significant, *F*(2,100) = 0.415, *p* = 0.662. There was no relative direct effect of the naïve condition on belief in CCCT: Controlling for distrust in climate science, participants in the naïve condition did not differ in terms of their belief in CCCT from the control group (*b* = 0.111, *p* = 0.691). The same was the case for the sophisticated group, which also did not have a relative direct effect on belief in CCCT (*b* = 0.194, *p* = 0.366). Also, both the relative indirect effects of the naïve condition (*b* = −0.262, 95% CI [−0.764, 0.247]) and the sophisticated condition (*b* = 0.179, 95% CI [−0.355, 0.736]) were not significant. Therefore, there was no mediation of the effect of the experimental conditions on belief in CCCT via distrust in climate science.

Furthermore, the index of moderated mediation was not significant, *b* = 0.121, 95% CI [−0.218, 0.521], indicating that political ideology did not moderate the effect of the experimental conditions on distrust in climate science.

### 3.3 Discussion

In Study 2, we attempted to establish a causal link between EBs and climate conspiracy beliefs. To achieve this, we randomly assigned participants to one of three conditions: a “naïve” EBs condition, a “sophisticated” EBs condition, and a control condition. However, the experimental manipulation only affected certainty beliefs, with participants in the “naïve” condition having significantly lower certainty beliefs than those in the “sophisticated” condition. All other EBs variables, and most importantly, belief in CCCT and distrust in climate science, were not affected by our experimental manipulation. Thus, the experimental groups did not differ in predicting distrust in climate science or belief in CCCT, and the predicted (moderated) mediation was not present. On the correlational level, however, we could replicate all relationships between the EBs’ subscales and distrust in climate science as well as belief in CCCT.

The experimental manipulation may have failed because participants could skip the page containing the text. The rather short reading times suggest this explanation. We think that some of the participants may have only read the first sentence, if at all, which begins as follows: “Knowledge about climate change is established and does not simply change” (naïve beliefs condition) or for the sophisticated beliefs condition: “Knowledge about climate change is still provisional and is constantly evolving.” The first sentence aimed to manipulate participants’ beliefs about certainty. Thus, the possibility that participants only read the first sentence or the presence of a primacy effect (Sullivan, 2019) might explain the partial success of the experimental manipulation. However, it is also possible that the manipulation texts were insufficient to induce changes in participants’ EBs. To address this issue, future studies could employ alternative manipulation strategies, such as targeting each dimension of EBs individually to make the manipulations more effective. Another explanation for the absence of an effect on belief in CCCT or distrust in climate science could be the fact that the study was slightly underpowered.

## 4 General discussion

We conducted two studies, one correlational and one experimental, to investigate whether EBs about climate change (e.g., beliefs about knowledge and knowing about climate change) significantly predicted belief in climate change conspiracy theories (CCCT). It provides correlational and – in contrast to previous work – also attempts to provide experimental evidence for the relationship between EBs and belief in CCCT while also illuminating the underlying mechanisms and conditions of this relationship. To measure EBs about climate change, we developed a scale based on Hofer and Pintrich’s (1997) four-dimensional framework (certainty, simplicity, source, justification of knowledge). However, confirmatory factor analysis revealed that a five-factor model – distinguishing between self and experts as sources of knowledge and focusing especially on subjective justification – provided a significantly better fit. Using this more nuanced measure, we identified significant correlations between the epistemic belief subscales, distrust in climate science, and belief in CCCT across both studies.

In study 1, we found that believing in the uncertainty of climate change knowledge and believing this knowledge should be justified through personal observations and gut feelings predicted greater belief in CCCT. Conversely, believing knowledge about climate change is complex and interconnected, as well as stemming from climate scientists, predicted lower belief in CCCT. The findings align with prior research, which links belief in conspiracy theories to views of knowledge as uncertain, intuition-based, or independent of evidence (Garrett and Weeks, 2017; Rudloff et al., 2022; Serrano et al., 2023). However, we did not find any significant relationships for the belief that knowledge originates from one’s own reasoning (cf. Serrano et al., 2023), which may be attributable to the low reliability of this subscale in our study. Therefore, future studies on this topic would benefit from using a different, more reliable scale for measuring EBs according to the Hofer and Pintrich (1997) framework. We think that the present study provides relevant groundwork for this. Additionally, our findings extend prior research by demonstrating that beliefs about the structure of knowledge are linked to conspiracy beliefs, a dimension of EBs that has not been previously examined in the context of conspiracy beliefs. Unlike prior studies that used various conceptualizations of EBs, we employed a slightly adapted version of the framework by Hofer and Pintrich (1997). Finding that this relationship persists across different theoretical conceptualizations and models suggests that EBs may serve as a robust predictor of conspiracy beliefs, regardless of the theoretical framework. This is not surprising, given that the existing theoretical frameworks share some similarities^4^ – faith in intuition, for example, is reflected in Hofer and Pintrich’s (1997) naïve justification beliefs, while the need for evidence corresponds to rather sophisticated justification beliefs. Accordingly, multiplist beliefs (i.e., perceiving knowledge as subjective, changeable, and self-constructed) reflect a combination of rather sophisticated^5^ EBs in the Hofer and Pintrich (1997) framework. Moreover, research has shown that EBs are related to various forms of conspiracy ideation, including general conspiracy theories (Garrett and Weeks, 2017), specific conspiracy theories about COVID-19 (Rudloff et al., 2022; Serrano et al., 2023), and climate change (this study). These findings suggest that EBs may influence conspiracy thinking across domains. Future research should explore whether EBs are also linked to conspiracy mentality, providing deeper insight into the cognitive foundations of conspiratorial thinking.

We also found that all relationships between the EBs’ subscales and belief in CCCT were mediated by (dis-)trust in climate science. Specifically, distrust in climate science increased belief in CCCT, while trust decreased it. Our results replicate the established links between EBs and trust in science (e.g., Strømsø et al., 2011; Schoor, 2023) and between trust in science and conspiracy beliefs (e.g., Sarathchandra and Haltinner, 2021b). Additionally, our study aligns with Serrano et al. (2023) by demonstrating that distrust in science mediates the relationship between EBs and conspiracy beliefs.

Conspiracy beliefs are inherently “oppositional” by definition (Douglas and Sutton, 2023, p. 282), which may help explain the observed association between such beliefs and distrust in science. But why is it that people’s EBs are related to their amount of trust in scientists? It is plausible that individuals who perceive scientific claims about climate as tentative or provisional may question the motives behind updates or changes, suspecting hidden agendas or biases, thereby fostering mistrust in climate science. Similarly, those who believe that knowledge should be based on personal observation and reasoning may regard scientists as untrustworthy, whereas individuals who view climate knowledge as derived from experts are more likely to trust scientific authorities. Nonetheless, these relationships could be bidirectional, highlighting the necessity for experimental and longitudinal studies to establish causality.

Contrary to our expectations, political ideology only moderated the relationship between belief in experts as a source of knowledge about climate change and distrust in climate science. The relationships between distrust and the other EBs dimensions were not dependent on political ideology. On the one hand, this may suggest that the other EBs dimensions themselves are sufficient to elicit trust or distrust in climate science. On the other hand, the absence of a moderating effect for these variables might be attributable to the low variability of political ideology in our sample. Content-wise, the moderation we found indicates that participants of the political center had the strongest negative relationship between expert beliefs and distrust in science compared to left-leaning participants, who had a weaker relationship. It is possible that for left-leaning participants, their trust in climate science is already high and less influenced by their belief in experts, possibly due to their ideological alignment with the scientific consensus on environmental issues. Supporting this interpretation is the significant positive relationship between political ideology and distrust in climate science (*r* = 0.51, *p* < 0.001), where higher values of political ideology correspond to the right spectrum – which also aligns with findings by scholars like Cologna et al. (2024) or Serrano et al. (2023).

In Study 2, we aimed to experimentally manipulate EBs. However, our experimental manipulation did not succeed, which is why we cannot provide any evidence for the causal direction of the relationship between EBs and conspiracy beliefs. This is why it is necessary to find and employ more effective ways to experimentally manipulate EBs. Moreover, it would be useful to consider alternative experimental designs to establish causality between the distinct components of our mediation model (Pirlott and MacKinnon, 2016). However, cross-sectionally, we were able to replicate the correlations between EBs, distrust in climate scientists, and belief in CCCT found in Study 1, which further substantiates the findings of the present research.

Taken together, our findings corroborate the notion that EBs play a significant role in the endorsement of conspiracy beliefs and illuminate the mechanisms underlying and shaping this association. Additionally, our results support the idea that conspiracy beliefs arise from epistemic, existential, and social motives (Douglas et al., 2017). On the one hand, EBs are involved in the urge to understand one’s environment (i.e., epistemic motives). As Gjoneska (2021) noted, analytic thinking, critical thinking, and scientific reasoning are “essential for reliable interpretation of events, and making sense of one’s environment” (p. 4) and impact belief in conspiracy theories. EBs, which are closely linked to how individuals reason about science and handle contradictory evidence, therefore can help to critically assess unwarranted claims and resist unfounded conspiracy narratives. On the other hand, our findings support the involvement of social motives in the development of conspiracy beliefs (Douglas et al., 2017), as distrust in climate science and scientists might reflect dissatisfied social motives (i.e., feeling connected to a group and having a positive image of one’s group). However, more experimental and longitudinal designs are needed to deepen our understanding of this motive-based notion (for an exception, see Liekefett et al., 2021).

### 4.1 Limitations

Despite the insights gained from this study, it is important to acknowledge its limitations. First, our data were collected only in Germany, which restricts our findings only to a WEIRD (Western, Educated, Industrialized, Rich, and Democratic; Henrich et al., 2010) population. This has theoretical implications, for example, regarding trust in science. While trust in scientists is higher among the political left overall, this relationship varies between countries and is not evident or even reversed in some countries (Cologna et al., 2024; preprint). Therefore, the moderating effect of political ideology that we found on the relationship between EBs and trust in science might differ when examined in other countries. With regard to the impact of political ideology on distrust, it is important to note that in both samples, participants’ political views were predominantly centered or left-leaning. In a sample with more right-leaning participants, the moderating effect of political ideology might be more pronounced, particularly among European or North American individuals. Moreover, we used a unidimensional measure of political ideology ranging from left to right. Such unidimensional conceptualizations of political ideology have been challenged because people can interpret the meaning of left and right differently (Bauer et al., 2017). At the same time, how these dimensions are understood differs between countries or cultures (Zechmeister, 2015; Hsiao et al., 2017). These variations in associations with left and right can affect the validity of political ideology measurements (Bauer et al., 2017). An alternative to this unidimensional approach is the use of multidimensional measures that include cultural and economic dimensions (Feldman and Johnston, 2014; Alves and Porto, 2022). However, we chose the ALLBUS left–right self-placement measure from the German General Social Survey, as our data were collected in Germany, where left and right are commonly used terms that simplify the concept of political ideology. Although the generalizability of our findings beyond German society is limited, it is noteworthy that a significant portion of our data was collected from non-student participants in the second study (with 76% students in Study 1 and 39% students in Study 2). This enables some generalization beyond the university student population, extending our findings to a broader group, although the sample remains non-representative.

Finally, we want to clarify that, after careful consideration, we have decided to make some deviations from our preregistration. This includes modifications to the first hypothesis and changes to the sample size in the second study. While it is common for researchers in psychological science to deviate from their preregistrations (Claesen et al., 2021; Willroth and Atherton, 2024), we believe that being transparent about these changes is essential for maintaining the credibility and replicability of the research. Therefore, the details of the deviations, along with their justifications and potential implications, are documented in our repository on the Open Science Framework (OSF; Foster and Deardorff, 2017).

### 4.2 Implications

The study highlights the crucial role of EBs and trust in science in shaping belief in climate change conspiracy theories. Theoretically, it deepens our understanding of the cognitive and trust-based mechanisms underlying conspiracy thinking. Practically, it suggests strategies for improving science communication, education, and policy to foster EBs that reduce distrust in climate science and conspiracy beliefs.

Effective science communication may help shape EBs in real-world settings by promoting beliefs that support the scientific approach while diminishing those that challenge it. To counteract perceptions of climate knowledge as uncertain, scientists and communicators should avoid emphasizing uncertainty in their messaging—even though uncertainty is undoubtedly a core value of science (Clegg, 2010). As the perceived certainty of climate change messages influences their perceived plausibility (Lombardi et al., 2014), communicating the stability of findings about climate change may also affect EBs about the certainty of climate change knowledge. Correspondingly, it is necessary to avoid false-balance media reports (i.e., balancing scientific consensus with contrarian voices in media reports) as they can lower the perception of a scientific consensus about climate change (Cook et al., 2017).

In addition to science communication, another cornerstone for changing EBs is education, particularly in secondary education. To address the reliance on personal observation in justifying climate change knowledge, educational initiatives can incorporate simple, relatable experiments that demonstrate concepts like the greenhouse effect and the water cycle. These hands-on activities connect scientific principles to everyday experiences, satisfying the need for personal observation while making scientific explanations more accessible and appealing. This idea is supported by a study by Xie et al. (2023), who found that participating in informal scientific activities is related to EBs that value scientific methods to justify knowledge. Moreover, personal justification can also be reduced through interventions that involve critical engagement with conflicting information from multiple sources about a scientific topic (Ferguson and Bråten, 2013). At the same time, such interventions can heighten the justification through science. Another approach to strengthening or supporting the development of EBs about science as a source of knowledge could involve educational initiatives aimed at enhancing science literacy. Science literacy can be defined as knowledge of scientific concepts, theories, and methods used to prove scientific hypotheses empirically. It is, therefore, associated with the ability to interpret and evaluate (pseudo-) scientific evidence (OECD, 2017). There is ample evidence that science literacy can be actively improved, for example, through participation in science courses (Surpless et al., 2014; Mahler et al., 2021) or through hands-on, problem-based learning in middle school classrooms (Lawless and Brown, 2015; Vieira and Tenreiro-Vieira, 2016). EBs are also positively related to science literacy (Wang et al., 2022), which is why we believe there is potential to foster pro-science EBs by enhancing science literacy. Finally, education predicts reduced belief in simple solutions to complex problems (Van Prooijen et al., 2015; van Prooijen, 2017) – which again is linked to conspiracy belief. Therefore, we argue that education can be a solution to enhance EBs about the complex structure of knowledge. On the theoretical level, one can, therefore, argue that EBs may also be a driving factor for the relationship between education and conspiracy theories (van Prooijen, 2017; Muscat-Inglott, 2023).

Our findings, combined with prior research on strategies to influence EBs, underscore the need for education-focused policies. These include teacher training, adult education programs, initiatives to enhance science literacy, and support for museums or informal learning environments, as previously recommended in a report for the European Parliament’s Committee on Culture and Education (Siarova et al., 2019).

## 5 Conclusion

In summary, our studies enhance the understanding of the conditions under which EBs about the nature of knowledge and knowing are connected to conspiracy beliefs about climate change. We discovered that EBs emphasizing uncertainty and personal justification of knowledge are associated with a higher endorsement of climate change conspiracy theories, while beliefs in the complexity and expert-driven nature of climate knowledge are associated with lower conspiracy beliefs. These relationships are mediated by trust in climate science, highlighting the crucial role that trust plays in shaping conspiracy ideation.

Theoretically, our results suggest that EBs shape how individuals engage with conspiracy narratives. Practically, the findings highlight the importance of improving science communication and educational strategies to foster EBs that promote scientific reasoning. Furthermore, our results support Schoor’s (2023) argument to reconceptualize what constitutes sophisticated EBs in a societal context. Ultimately, fostering EBs that embrace scientific principles and strengthening trust in experts are vital strategies for combating climate change conspiracy beliefs.

## Data Availability

The datasets presented in this study can be found in online repositories. The names of the repository/repositories and accession number(s) can be found at: https://osf.io/5v76b (Study 1) + https://osf.io/q2djt (Study 2).

## Footnotes

^1^Next to the term “climate conspiracy beliefs” (e.g., Bertin et al., 2021; Biddlestone et al., 2022), other constructs have been used to describe how people perceive and respond to information about climate change [e.g., “belief in climate change” (Hornsey et al., 2016); “climate skepticism” (Hornsey et al., 2018); “rejection of climate science” (Lewandowsky et al., 2013)].

^2^For a more detailed overview of this topic, see Sinatra et al. (2014).

^3^This conception of epistemic beliefs is similar to Hofer and Pintrich’s (1997) “justification” dimension but adds a new component, namely the idea of truth being shaped by political processes.

^4^Although the conceptions of epistemic beliefs employed in the studies by Garrett and Weeks (2017), Rudloff et al. (2022), and Serrano et al. (2023) do not differ extremely, their measurement, however, differs considerably. Garrett and Weeks (2017) and Rudloff et al. (2022) measured “general” epistemic beliefs, while Serrano et al. (2023) and we employed subject-specific measures (i.e., beliefs about knowledge about COVID-19, or, in our case, climate change).

^5^However, what is seen as sophisticated according to the framework depends on its scientific context (Elby and Hammer, 2001; Sinatra et al., 2014). For example, the idea of “tentative knowledge” is often perceived as sophisticated, even though it may not be sophisticated when speaking about the consensus of human-made climate change. Similarly, excessively trusting experts is not seen as sophisticated, whereas actively gaining knowledge through reasoning is seen as rather sophisticated. Following Sinatra et al. (2014), we assert that trusting experts as reliable sources of knowledge is not inherently naïve, especially when the information is scientifically grounded and supported by empirical evidence (see also Schoor, 2023).
