# Supplementary material for: How Epistemic Beliefs about Climate Change Predict Climate Change Conspiracy Beliefs
Source: Front Psychol. 2025 Apr 14;16:1523143. doi: 10.3389/fpsyg.2025.1523143 (PMC12035771; doi:10.3389/fpsyg.2025.1523143)
Supplement: Supplementary file 1 [file Supplementary_file_1.docx]

Supplementary Material

# Confirmatory Factor Analysis (CFA) – Study 1

We conducted a CFA in order to test whether our items loaded on the four factors of the Hofer and Pintrich (1997) framework (certainty, simplicity, source, justification of knowledge). The CFA was carried out in R, using the package lavaan (Rosseel, 2012). We checked whether our values were missing completely at random (Little’s-test), and checked for multi- and univariate normality (Mardia’s-test and Shapiro-Wilk-test).

Little’s test was not significant, χ²(1011) = 1054, *p* = .169, indicating that the values are missing completely at random (MCAR). Mardia’s-test was significant for skewness and (*p* < .001) and kurtosis (*p* = 0), and Shapiro-Wilk test was significant for all items (*n* = 24, *p* < .001). As our data were not normally distributed, we used a robust maximum likelihood estimator (MLR; robust Huber-White standard errors and scaled test statistic asymptotically equal to the Yuan-Bentler test statistic). Our missing values were MCAR, which is why we used Full information maximum likelihood (FIML) to handle the missing values. Lastly, we fixed the variances of the latent factors to 1.

To evaluate whether our data align with the proposed four-factor structure, we used the following fit indices: Yuan-Bentler scaled chi-square test statistic (*χ²*/df ≤ 2 or 3; Schreiber et al., 2006), comparative fit index (CFI ≥ .95), root mean square error of approximation (RMSEA < .06), and standardized root mean square residuals (SRMR < .08) (Hu & Bentler, 1999).

The four-factor structure yielded the following robust fit indices: *χ²* (246) = 1191.48, *p* < .001, CFI = .547, RMSEA = .102, SRMR = .124. The fit indices suggest that the model does not fit the data. Thus, we inspected the estimates for the factor loadings, the covariances between the latent factors, and the modification indices (cross-loadings; correlated error terms). However, the model fit did not significantly improve despite several modifications, including removing items with factor loadings below 0.50 and above 1.00, removing items that exhibited cross-loadings, and adding correlated error terms between items as suggested by the modification indices.

As a final measure, we examined the factor structure using Exploratory Factor Analysis (EFA, Table S2). First of all, we removed items with cross-loadings (*Certatiny1, Certainty5, Acquisition4, Justification2, Justification3*) and loadings below .30 (*SimplicityContext3*). Next, we assessed which sets of items were associated with each factor. We found that the following items loaded on Factor 1:

1. *Certainty6:* “There are certain facts in climate research that are generally recognized and undisputed.”^[[1]](#footnote-1)^
2. *SimplicityContext6*: “There are clear facts in climate science that are independent of context.”
3. *Acquistion5:* “To really understand the climate, you should rely on the knowledge of experts.”
4. *Acquisition6:* “Experts are the main source of reliable knowledge about the climate.”
5. *Justification1:* “Information about the climate is more reliable if it is based on scientific methods.”

Although we recognize that the first two items align with the factor’s general theme, we believe that they assess different aspects (namely the “certainty of facts”), compared to the last three items that focus on expert or scientific knowledge. Moreover, the first two items of this factor exhibited comparably low factor loadings. Thus, we excluded the items *Certainty6* and *SimplicityContext6.*

The following items loaded on Factor 2:

1. *Simplicity1:* “Knowledge about the climate consists of many interrelated facts.”
2. *Simplicity2:* “To understand climate science, you have to recognize how different concepts are connected.”
3. *Simplicity4:* “Climate science is mainly based on isolated, unconnected facts.”
4. *Simplicity5:* “There are few connections between different pieces of information in climate science.”

The items capture the structure of knowledge about climate change. However, the second item exhibits a low factor loading, which is why we excluded it as well.

Factor 3 consisted of the following items:

1. *Certainty2:* “New findings can call previous assumptions of climate science into question.”
2. *Certainty3:* “Knowledge about the climate is constantly changing.”
3. *Certainty4:* “The findings of climate science are certain and do not change.”

All items load substantially on the factor and measure whether one considers climate knowledge as variable or fixed.

The following items loaded on Factor 4:

1. *Acquisition1*: “To understand the climate, you have to actively and personally engage with the topic.”
2. *Acquisition2:* “Engaging with others plays an important role in understanding the climate.”
3. *Acquisition3:* “Doing your own research is important to understand the climate.”

The items relate to playing an active role in gaining knowledge about the climate. All items load substantially on the factor.

The last factor comprises the following items:

1. *Justification4:* “Knowledge about the climate is often based on personal observations and experiences.”
2. *Justification5:* “Gut feeling often plays a role when judging information about the climate.”
3. *Justification6:* “Personal anecdotes and stories are often more convincing than statistical data on the climate.”

These items load substantially on Factor 5. They capture a more subjective approach in understanding and evaluating climate-related information.

We then run a CFA with this particular selection of items and tested whether the following model was accurately representing the factor structure of our data^[[2]](#footnote-2)^:

Certainty =~ Certainty2 + Certainty3 + Certainty4

Simplicity =~ Simplicity1 + Simplicity4 + Simplicity5

Acquisition_own =~ Acquisition1 + Acquisition2 + Acquisition3

Acquisition_expert =~ Acquisition5 + Acquisition6 + Justification1

Justification_subjective =~ Justification4 + Justification5 + Justification6

The five-factor structure yielded the following robust fit indices: *χ²* (80) = 176.66, *p* < .001, CFI = .911, RMSEA = .057, SRMR = .057.

This model had overall better fit indices than the original model. However, there were significant covariances between some of the latent factors and the modification indices suggested correlated error terms between the items Simplicity4 and Simplicity5. Therefore, we added the following correlations to the model^[[3]](#footnote-3)^:

Simplicity ~~ Acquisition_expert

Simplicity ~~ Justification

Acquisition_own ~~ Justification

Acquisition_expert ~~ Justification

Simplicity4 ~~ Simplicity5

The following fit indices were obtained for this adjusted model: *χ²* (79) = 165.16, *p* < .001, CFI = .921, RMSEA = .054, SRMR = .056. The model with the corelations between the latent factors and the items Simplicity4 and Simplicity5 therefore had slightly improved fit indices. Overall, the model demonstrated an acceptable to good fit to the data. The Chi-Square-test was significant, however, as the test is sensitive to sample size, we also looked at the ratio of *χ²* to df (*χ²*/df), which was approximately 2.09 and thus below the threshold of 3, indicating an acceptable model fit. The CFI was slightly below the threshold of .95. The RMSEA was below its cut-off (.06), signifying a good fit. The SRMR was also below the cut-off (.08), indicating acceptable fit.

## Discussion

The initial set of items was designed to assess the four dimensions of Hofer and Pintrich’s (1997) epistemic beliefs framework. However, the proposed four-factor structure was not supported by our data. Through the elimination of certain items and the reallocation of others, we developed a revised scale that demonstrated an acceptable to good fit. The final set of items now measures the following five dimensions:

1. **Certainty of Knowledge** (i.e., knowledge is perceived as certain or uncertain),
2. **Structure of Knowledge** (i.e., knowledge is viewed as simple vs. complex and interrelated),
3. **Self as a Source of Knowledge** (i.e., personal effort is considered a source of knowledge),
4. **Experts as a Source of Knowledge** (i.e., experts and scientific authorities are regarded as sources of knowledge), and
5. **Subjective Justification** (i.e., knowledge is justified through intuition and personal observation).

The first two dimensions – *Certainty of Knowledge* and *Structure of Knowledge* – clearly align with Hofer and Pintrich’s (1997) original dimensions of “certainty of knowledge” and “simplicity of knowledge.” The third and fourth dimensions, *Self as a Source of Knowledge* and *Experts as a Source of Knowledge*, correspond to the two ends of Hofer and Pintrich’s (1997) “source of knowledge” dimension, which posits that knowledge can be actively constructed by individuals or derived from external authorities such as experts and scientific institutions. Additionally, the fourth dimension, *Experts as a Source of Knowledge*, aligns with one end of the “justification” dimension in Hofer and Pintrich’s framework, which asserts that knowledge should be justified through scientific evidence. Conversely, the fifth dimension, *Subjective Justification*, reflects the other end of the “justification” dimension, emphasizing the role of personal observations and intuition in justifying knowledge.

The emergence of a five-factor structure can be mainly attributed to the fact that we split up the “source” dimension by Hofer and Pintrich (1997). By distinguishing between *Self as a Source of Knowledge* and *Experts as a Source of Knowledge*, we acknowledge that these sources are not mutually exclusive. For instance, an individual who values scientific authorities can simultaneously engage in personal research and reasoning to deepen their understanding of a topic. This separation allows for a more detailed exploration of how personal initiative and reliance on scientific sources coexist, thereby providing a more nuanced assessment of epistemic beliefs. Moreover, we think that there is a conceptual overlap between the “source of knowledge” and “justification” dimensions in Hofer and Pintrich’s (1997) framework (Greene et al., 2008). It is likely that one’s beliefs about the source of knowledge influence the criteria one uses to justify that knowledge. For example, when individuals view experts as a source of knowledge, they are likely to adopt empirical standards for justifying that knowledge. Conversely, when knowledge is derived from personal experience or intuition, the justification may rely more on subjective criteria. This is why the items of our *Experts as a Source of Knowledge* dimension may overlap with Hofer and Pintrich’s (1997) notion of “justification by authority or experts”.

In summary, even though we originally developed the items to match the proposed four-factor structure, we think that the revised five-factor structure is helpful to capture the complexity of epistemic beliefs in a more nuanced way.

# Exploratory Factor Analysis (EFA) – Study 1

An EFA with promax rotation was performed in R-Studio, using the package psych (Revelle, 2024). To assess whether the conditions for EFA were met, the Kaiser–Meyer–Olkin (KMO) measure of sampling adequacy and the Bartlett’s test of sphericity were conducted. Multicollinearity was checked visually using an item-correlation-matrix. Previously, missing values were identified and excluded. To determine the number of factors, the following criteria were used: Eigenvalues > 1.0 (Kaiser's criterion), sharp descent in the scree plot’s curve, and Eigenvalues greater than the mean Eigenvalues from a parallel analysis. Factor loadings > .40 were considered substantively important (Field et al., 2012).

An exploratory factor analysis was conducted using the 24 epistemic beliefs items. After removing missing values, the sample consisted of *n* = 333 cases. There were no item-correlations greater than *r* = .90, suggesting the absence of multicollinearity (see Table S1). All values in the KMO-test were greater than *MSA* = .73 with a mean value of *MSA* = .81, indicating a good sampling adequacy (Kaiser, 1970). The Bartlett’s test was significant, with *χ*²(276) = 2189.07, *p* < .001, leading to the conclusion that the variables in the dataset were not uncorrelated. Thus, the conditions for running an EFA were given.

The scree plot and parallel analysis resulted in a five-factor model (Figure S1) and five Eigenvalues were greater than 1. This is why we fitted a five-factor model. A table with the items and their loadings on the five factors can be found in Table S2. One factor loading was below the cut-off of r <|0.30| (Simplicity context beliefs 3), but the other items loaded substantively on at least one factor. However, a few items displayed factor loadings smaller than .40. Some items loaded on more than one factor (Certainty beliefs items 1 and 5; Acquisition beliefs item 4; Justification beliefs items 2 and 3). The model accounts for 40 % of variance.

# References

Field, A. P., Miles, J., & Field, Z. (2012). *Discovering statistics using R*. Sage.

Greene, J. A., Azevedo, R., & Torney-Purta, J. (2008). Modeling Epistemic and Ontological Cognition: Philosophical Perspectives and Methodological Directions. *Educational Psychologist*, *43*(3), 142–160. https://doi.org/10.1080/00461520802178458

Hu, L., & Bentler, P. M. (1999). Cutoff criteria for fit indexes in covariance structure analysis: Conventional criteria versus new alternatives. *Structural Equation Modeling: A Multidisciplinary Journal*, *6*(1), 1–55. https://doi.org/10.1080/10705519909540118

Kaiser, H. F. (1970). A second generation little jiffy. *Psychometrika*, *35*(4), 401–415. https://doi.org/10.1007/BF02291817

Revelle, W. (2024). *psych: Procedures for Psychological, Psychometric, and Personality Research* (Version 2.4.3) [Computer software]. https://cran.r-project.org/web/packages/psych/index.html

Rosseel, Y. (2012). lavaan: An *R* Package for Structural Equation Modeling. *Journal of Statistical Software*, *48*(2). https://doi.org/10.18637/jss.v048.i02

Schreiber, J. B., Nora, A., Stage, F. K., Barlow, E. A., & King, J. (2006). Reporting Structural Equation Modeling and Confirmatory Factor Analysis Results: A Review. *The Journal of Educational Research*, *99*(6), 323–338. https://doi.org/10.3200/JOER.99.6.323-338

# Table S1

|  | 1 | 2 | 3 | 4 | 5 | 6 | 7 | 8 | 9 | 10 | 11 | 12 | 13 | 14 | 15 | 16 | 17 | 18 | 19 | 20 | 21 | 22 | 23 |
| --- | --- | --- | --- | --- | --- | --- | --- | --- | --- | --- | --- | --- | --- | --- | --- | --- | --- | --- | --- | --- | --- | --- | --- |
| 1. Cert1 | 1 |  |  |  |  |  |  |  |  |  |  |  |  |  |  |  |  |  |  |  |  |  |  |
| 1. Cert2 | .23 | 1 |  |  |  |  |  |  |  |  |  |  |  |  |  |  |  |  |  |  |  |  |  |
| 1. Cert3 | .27 | .39 | 1 |  |  |  |  |  |  |  |  |  |  |  |  |  |  |  |  |  |  |  |  |
| 1. Cert4_r | .15 | .36 | .37 | 1 |  |  |  |  |  |  |  |  |  |  |  |  |  |  |  |  |  |  |  |
| 1. Cert5_r | -.09 | .18 | .26 | .39 | 1 |  |  |  |  |  |  |  |  |  |  |  |  |  |  |  |  |  |  |
| 1. Cert6_r | -.28 | .11 | .22 | .23 | .41 | 1 |  |  |  |  |  |  |  |  |  |  |  |  |  |  |  |  |  |
| 1. Simpl1 | .41 | .17 | .02 | .03 | -.11 | -.21 | 1 |  |  |  |  |  |  |  |  |  |  |  |  |  |  |  |  |
| 1. Simpl2 | .26 | .22 | .14 | .07 | -.01 | -.09 | .40 | 1 |  |  |  |  |  |  |  |  |  |  |  |  |  |  |  |
| 1. SimplCon3 | .03 | .18 | .31 | .20 | .33 | .27 | -.04 | .10 | 1 |  |  |  |  |  |  |  |  |  |  |  |  |  |  |
| 1. Simpl4_r | .18 | .03 | -.14 | .09 | -.05 | -.17 | .35 | .22 | -.15 | 1 |  |  |  |  |  |  |  |  |  |  |  |  |  |
| 1. Simpl5_r | .16 | .05 | -.14 | .04 | -.13 | -.21 | .33 | .17 | -.14 | .40 | 1 |  |  |  |  |  |  |  |  |  |  |  |  |
| 1. SimplCon6_r | -.06 | .03 | .09 | .23 | .21 | .36 | -.21 | -.07 | .28 | -.04 | -.16 | 1 |  |  |  |  |  |  |  |  |  |  |  |
| 1. Acquisition1 | .15 | .08 | .14 | .14 | .14 | .01 | .18 | .29 | .16 | .03 | .08 | -.02 | 1 |  |  |  |  |  |  |  |  |  |  |
| 1. Acquisition2 | .06 | .14 | .08 | .04 | .02 | -.05 | .18 | .17 | .18 | .01 | .05 | .03 | .31 | 1 |  |  |  |  |  |  |  |  |  |
| 1. Acquisition3 | .05 | .10 | .16 | .12 | .15 | .14 | .04 | .10 | .13 | -.03 | -.07 | .04 | .38 | .31 | 1 |  |  |  |  |  |  |  |  |
| 1. Acquisition4_r | -.23 | -.04 | .01 | .08 | .20 | .20 | -.18 | -.14 | .10 | .04 | .02 | .21 | -.12 | -.05 | -.09 | 1 |  |  |  |  |  |  |  |
| 1. Acquisition5_r | -.34 | -.07 | .09 | .07 | .23 | .37 | -.36 | -.23 | .18 | -.15 | -.22 | .25 | -.20 | -.06 | -.06 | .46 | 1 |  |  |  |  |  |  |
| 1. Acquisition6_r | -.29 | -.18 | .11 | .12 | .20 | .29 | -.35 | -.23 | .21 | -.24 | -.18 | .22 | -.07 | -.01 | -.01 | .40 | .60 | 1 |  |  |  |  |  |
| 1. Justi1 | .24 | .05 | -.14 | -.15 | -.26 | -.26 | .31 | .25 | -.27 | .26 | .24 | -.32 | .03 | -.06 | -.05 | -.33 | -.54 | -.50 | 1 |  |  |  |  |
| 1. Justi2 | .35 | .23 | .16 | .24 | .11 | -.02 | .39 | .33 | .18 | .13 | .15 | -.06 | .31 | .31 | .25 | -.15 | -.12 | -.08 | .15 | 1 |  |  |  |
| 1. Justi3 | .29 | .17 | .02 | .16 | -.05 | -.18 | .45 | .39 | .00 | .26 | .27 | -.19 | .23 | .15 | .10 | -.20 | -.37 | -.25 | .35 | .49 | 1 |  |  |
| 1. Justi4_r | .14 | .02 | -.20 | -.05 | -.18 | -.15 | .23 | .06 | -.32 | .27 | .21 | -.10 | -.06 | -.13 | -.09 | -.16 | -.17 | -.24 | .26 | .04 | .13 | 1 |  |
| 1. Justi5_r | .07 | -.02 | -.08 | -.03 | -.05 | -.02 | .11 | .10 | -.18 | .16 | .17 | -.09 | -.02 | -.11 | -.04 | -.17 | -.16 | -.08 | .15 | .07 | .12 | .38 | 1 |
| 1. Justi6_r | .03 | .06 | -.10 | .05 | -.03 | -.01 | .16 | .05 | -.18 | .11 | .20 | -.07 | .00 | -.13 | -.07 | -.27 | -.17 | -.15 | .19 | .08 | .17 | .44 | .37 |

Note. *Cert** is an abbreviation for certainty beliefs, *Simpl** is an abbreviation for simplicity beliefs, *SimplCon** is an abbreviation for context-specific simplicity beliefs, *Acquisition** is an abbreviation for acquisition beliefs, *Justi** is an abbreviation for justification beliefs.

# Figure S1


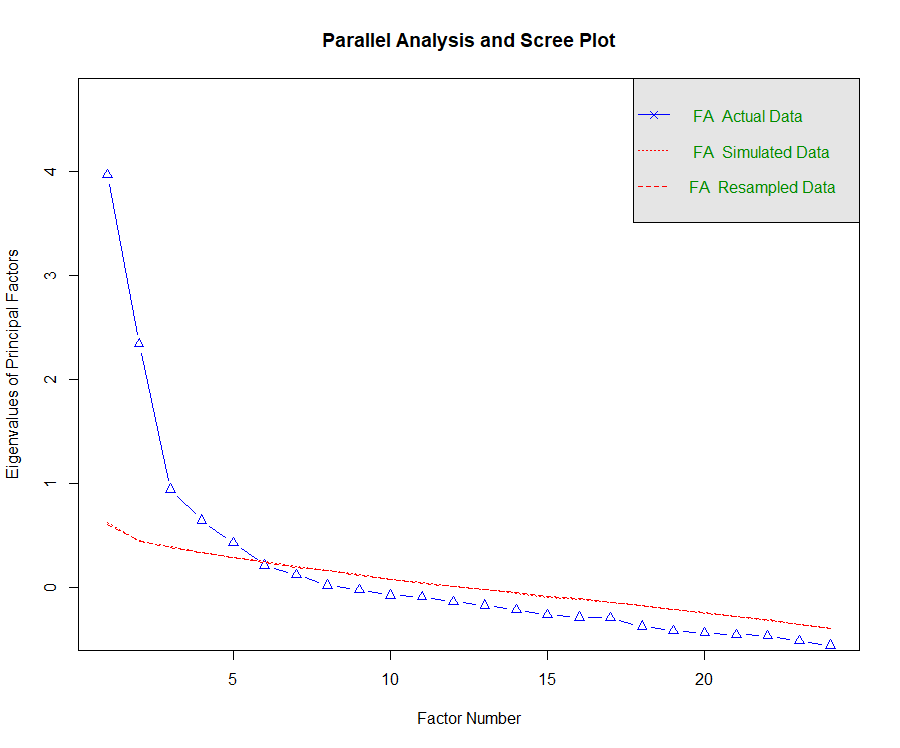


**Supplementary Figure S1.** Scree Plot and Parallel Analysis of the Exploratory Factor Analysis (EFA).

# Table S2

| **Item-Text** | **Item Code** | **Factor 1** | **Factor 2** | **Factor 3** | **Factor 4** | **Factor 5** |
| --- | --- | --- | --- | --- | --- | --- |
| 1. Das Wissen in der Klimawissenschaft entwickelt sich ständig weiter. | Cert1 | -.40 |  | .38 |  |  |
| 1. Neue Forschungsergebnisse können bisherige Annahmen in der Klimawissenschaft in Frage stellen. | Cert2 |  |  | .65 |  |  |
| 1. Das Wissen über das Klima ändert sich ständig. | Cert3 |  |  | .70 |  |  |
| 1. Die Erkenntnisse der Klimawissenschaft stehen fest und ändern sich nicht. | Cert4 |  |  | .60 |  |  |
| 1. Forschende sind sich über die Grundlagen der Klimawissenschaft vollkommen einig. | Cert5 | .30 |  | .38 |  |  |
| 1. Es gibt in der Klimaforschung bestimmte Fakten, die allgemein anerkannt und unumstritten sind. | Cert6 | .37 |  |  |  |  |
| 1. Das Wissen über das Klima besteht aus vielen Fakten, die miteinander zusammenhängen. | Simpl1 |  | .55 |  |  |  |
| 1. Um die Klimawissenschaft zu verstehen, muss man erkennen, wie verschiedene Konzepte zusammenhängen. | Simpl2 |  | .33 |  |  |  |
| 1. Klimawissen hängt stark vom Kontext ab, in dem es betrachtet wird. | SimplCon3 |  |  |  |  |  |
| 1. Klimawissenschaft basiert hauptsächlich auf einzelnen, unverbundenen Fakten. | Simpl4 |  | .67 |  |  |  |
| 1. Es gibt wenige Zusammenhänge zwischen den verschiedenen Informationen in der Klimawissenschaft. | Simpl5 |  | .60 |  |  |  |
| 1. Es gibt in der Klimawissenschaft klare Fakten, die unabhängig vom Kontext sind. | SimplCon6 | .31 |  |  |  |  |
| 1. Das Verstehen des Klimas erfordert aktive Beteiligung und persönliche Auseinandersetzung mit dem Thema. | Acquisition1 |  |  |  | .60 |  |
| 1. Der Austausch mit anderen spielt eine wichtige Rolle beim Verstehen des Klimas. | Acquisition2 |  |  |  | .56 |  |
| 1. Eigene Recherchen sind wichtig, um das Klima zu verstehen. | Acquisition3 |  |  |  | .55 |  |
| 1. Wissen über das Klima kann am besten von Expert*innen auf diesem Gebiet vermittelt werden. | Acquisition4 | .72 | .39 |  |  |  |
| 1. Um das Klima wirklich zu verstehen, sollte man sich auf das Wissen von Expert*innen stützen. | Acquisition5 | .80 |  |  |  |  |
| 1. Fachleute sind die Hauptquelle für verlässliches Wissen über das Klima. | Acquisition6 | .74 |  |  |  |  |
| 1. Informationen über das Klima sind zuverlässiger, wenn sie auf wissenschaftlichen Methoden beruhen. | Justi1 | -.56 |  |  |  |  |
| 1. Bei Aussagen über das Klima ist es wichtig, verschiedene Informationsquellen miteinander zu vergleichen und zu verknüpfen. | Justi2 |  | .35 |  | .57 |  |
| 1. Es ist entscheidend, bei Informationen über das Klima die Qualität und Zuverlässigkeit der Quellen zu bewerten. | Justi3 |  | .42 |  | .36 |  |
| 1. Wissen über das Klima basiert oft auf persönlichen Beobachtungen und Erfahrungen. | Justi4 |  |  |  |  | .51 |
| 1. Oft spielt das Bauchgefühl eine Rolle bei der Bewertung von Informationen über das Klima. | Justi5 |  |  |  |  | .51 |
| 1. Persönliche Anekdoten und Geschichten sind oft überzeugender als Statistiken zum Klima. | Justi6 |  |  |  |  | .71 |

Note. *Cert** is an abbreviation for certainty beliefs, *Simpl** is an abbreviation for simplicity beliefs, *SimplCon** is an abbreviation for context-specific simplicity beliefs, *Acquisition** is an abbreviation for acquisition beliefs, *Justi** is an abbreviation for justification beliefs.

# Table S3

*Experimental Manipulation (study 2)*

|  | Original (German) | Translation |
| --- | --- | --- |
| Group 1: Naïve beliefs | Das Wissen über den Klimawandel steht fest und ändert sich nicht einfach so. Es besteht aus einzelnen Fakten, die für sich alleinstehen und unabhängig vom Kontext sind. Damit man das Klima wirklich verstehen kann, ist es wichtig, sich auf die umfassenden Erkenntnisse und Erklärungen von echten Fachleuten zu stützen. Um dann herauszufinden, ob Behauptungen über das Klima wahr sind, verlassen sich viele Menschen auf eigene Beobachtungen, Meinungen von Fachleuten oder ihr Bauchgefühl. […]  (72 words) | Knowledge about climate change is established and does not simply change. It consists of individual facts that stand alone and are independent of context. To truly understand the climate, it is important to rely on the comprehensive findings and explanations of real experts. To find out whether claims about the climate are true, many people rely on their own observations, the opinions of experts or their gut feeling. […] |
| Group 2: Sophisticated beliefs | Das Wissen über den Klimawandel ist noch vorläufig und entwickelt sich immer weiter. Es besteht aus Konzepten, die miteinander zusammenhängen und abhängig vom Kontext sind. Damit man das Klima wirklich verstehen kann, ist es wichtig, sich selbst aktiv damit zu beschäftigen und mit anderen darüber zu sprechen. Um dann herauszufinden, ob Behauptungen über das Klima wahr sind, stellen viele Menschen eigene Nachforschungen an oder lesen und bewerten unterschiedliche Quellen. […]  (69 words) | Knowledge about climate change is still provisional and is constantly evolving. It consists of concepts that are interrelated and dependent on context. To truly understand the climate, it is important to actively engage with it and talk about it with others. To find out whether claims about the climate are true, many people do their own research or read and evaluate different sources. [...] |
| Control Group | No text | No text |

1. Translated from the German wording of the item. All following items were also translated from German. [↑](#footnote-ref-1)
2. =~ means “is measured by” [↑](#footnote-ref-2)
3. ~~ means “is correlated with” [↑](#footnote-ref-3)
